# Detailed data about a forty-year systematic review and meta-analysis on nursing student academic outcomes

**DOI:** 10.1016/j.dib.2021.107298

**Published:** 2021-08-14

**Authors:** Valeria Caponnetto, Angelo Dante, Vittorio Masotta, Carmen La Cerra, Cristina Petrucci, Celeste Marie Alfes, Loreto Lancia

**Affiliations:** a Department of Health, Life and Environmental Sciences, University of L'Aquila, Rita Levi Montalcini Building, G. Petrini Street - 67100, L'Aquila, Italy; b Neuroscience Section, Department of Applied Clinical Sciences and Biotechnology, University of L'Aquila, Vetoio Street - 67100, L'Aquila, Italy; c Frances Payne Bolton School of Nursing, Case Western Reserve University, Health Education Office 269B, Cleveland, Ohio, USA

**Keywords:** Academic failure, Academic success, Attrition, Bachelor's degree, Determinants, Factors, Nursing student

## Abstract

Data were extracted from observational studies describing undergraduate nursing students’ academic outcomes that were included in a systematic review and meta-analysis conducted in 2019 and updated in 2020 [1]. Data were extracted by two researchers independently through a previously tested electronic spreadsheet; any disagreement about data extraction was discussed with a third author. Extracted data were studies’ general information, characteristics (i.e., country, study design, involved centers, number of cohort of students involved, duration (years) and denomination of the program attended, sample (*N*), sociodemographic characteristics of the sample, and methods utilized for data collection), and data related to the research question(s) of the review, i.e., nursing students’ academic outcomes occurrence and associated factors. Raw data for each included study are reported, along with meta-analyses that were performed using ProMeta free software utilizing Odds Ratio (OR) and Cohen's *d* as principal effect sizes. The random-effect model was used for all studies, while the level of heterogeneity was explored and quantified through the Cochran's Q-test and *I^2^*, respectively. Substantial or considerable heterogeneity (i.e., *I^2^* ≥ 50%) was explored through a subgroup analysis based on the study design, when feasible [2]. A sensitivity analysis was also performed to detect the possible influence of single studies on meta-analyses results [2]. Publication bias was assessed through funnel plots and the testsf for their asymmetry, i.e., Begg and Mazumdar's rank correlation and Egger's linear regression method [2]. These data provide for an updated state of the art about nursing students’ outcomes and associated factors. Therefore, they could ease future literature summaries about the topic, other than allow a comparison of the literature with future research results.

## Specifications Table

**Table:** 

Subject	Nursing and Health Professions
Specific subject area	Associated factors of nursing students’ academic outcomes
Type of data	Tables Figures
How data were acquired	Data were acquired consulting the studies included in the systematic review. Included studies were retrieved launching search strings on PubMed, Scopus, Education Resources Information Centre (ERIC), and Open Grey databases. To maximize the finding of potentially relevant manuscripts, the reference lists of the included studies, as well as the references that had cited the included studies on Scopus were assessed for eligibility.
Data format	Raw Analysed
Parameters for data collection	Studies were included in the systematic review and data were extracted if studies were: available in full text, published in Italian or English languages, and quantitative non-randomized in design. Moreover, studies had to: include academic nursing students that attended a program lasting at least three years, consider the academic outcomes measured at the end of the regular duration of the program according to its regulation, and report an analysis of associated or predictive factors of students’ academic outcomes.
Description of data collection	After a two-step screening of abstracts and full texts according to relevant inclusion criteria, 18 studies (*n* =10 were retrospective cohort, *n* = 7 were prospective cohort, *n* =1 was and case-control) were included in the review. Nine studies were included in meta-analysis. The ‘Downs and Black instrument’ was used to assess the risk of bias of included studies [3]. Data were extracted and collected by two researchers independently after printing included full texts. Any disagreement between the two researchers about data extraction was solved by discussion with a third author. Data were inserted in an electronic spread sheet of Excel for Windows, where the following data were collected: first author, publication year, Country, study design, whether the study was multicentric, number of cohorts of students, program duration (years), program denomination, sample (*N*), number of females, age [Mean (SD)], methods for data collection, study aim, adopted definition of the outcome, independent variables assessed for the association with/prediction of the outcome and their occurrence or mean values, statistical analyses performed by the authors, and summary of results provided referred to all the outcome definitions adopted by the authors (if available).
Data source location	Raw data: the list of all retrieved references in all the electronic search strategies performed is provided. Moreover, all data extracted from included manuscripts in accordance with the systematic review protocol are provided in Excel for Windows spreadsheets. Analysed data Institution: University of L’Aquila City: L'Aquila Country: Italy Primary data sources: M.J. Brimble, Does entry route really affect academic outcome? Academic achievement of traditional versus non traditional entrants to BN (Hons) pre-registration nursing programmes. Journal of Further and Higher Education. 39 (2015) 379–398 G. Byrd, C. Garza, R. Nieswiadomy, Predictors of successful completion of a baccalaureate nursing program. Nurse Educ. 24 (1999) 33–37 https://doi.org/10.1097/00006223-199911000-00014. A. Dante, S. Fabris, A. Palese, Time-to-event analysis of individual variables associated with nursing students' academic failure: a longitudinal study. Adv Health Sci Educ Theory Pract. 18 (2013) 1047–1065 https://doi.org/10.1007/s10459-013-9448-6. A. Dante, S. Fabris, A. Palese, Predictive power of individual factors and clinical learning experience on academic success: Findings from a longitudinal study. Nurse educator. 40 (2015) E1–E6 A. Dante, G. Valoppi, L. Saiani, A. Palese, Factors associated with nursing students' academic success or failure: a retrospective Italian multicenter study. Nurse Educ Today. 31 (2011) 59–64 https://doi.org/10.1016/j.nedt.2010.03.016. I.J. Deary, R. Watson, R. Hogston, A longitudinal cohort study of burnout and attrition in nursing students. J Adv Nurs. 43 (2003) 71–81 https://doi.org/10.1046/j.1365-2648.2003.02674.x. M. Ehrenfeld, A. Rotenberg, R. Sharon, R. Bergman, Reasons for student attrition on nursing courses: a study. Nurs Stand. 11 (1997) 34–38 https://doi.org/10.7748/ns1997.02.11.23.34.c2443. J. Kevern, C. Ricketts, C. Webb, Pre-registration diploma students: a quantitative study of entry characteristics and course outcomes. J Adv Nurs. 30 (1999) 785–795 https://doi.org/10.1046/j.1365-2648.1999.01175.x. H.J. Knopke, Predicting student attrition in a baccalaureate curriculum. Nurs Res. 28 (1979) 224–227. L. Lancia, V. Caponnetto, A. Dante, A. Mattei, C. La Cerra, M.G. Cifone, C. Petrucci, Analysis of factors potentially associated with nursing students' academic outcomes: A thirteen-year retrospective multi-cohort study. Nurse Educ Today. 70 (2018) 115–120 https://doi.org/10.1016/j.nedt.2018.08.020. J. Mulholland, E.N. Anionwu, R. Atkins, M. Tappern, P.J. Franks, Diversity, attrition and transition into nursing. J Adv Nurs. 64 (2008) 49–59 https://doi.org/10.1111/j.1365-2648.2008.04758.x. V. Pitt, D. Powis, T. Levett-Jones, S. Hunter, The influence of personal qualities on performance and progression in a pre-registration nursing programme. Nurse Educ Today. 34 (2014) 866–871 https://doi.org/10.1016/j.nedt.2013.10.011. V. Pitt, D. Powis, T. Levett-Jones, S. Hunter, The influence of critical thinking skills on performance and progression in a pre-registration nursing program. Nurse Educ Today. 35 (2015) 125–131 https://doi.org/10.1016/j.nedt.2014.08.006. S. Pryjmachuk, K. Easton, A. Littlewood, Nurse education: factors associated with attrition. J Adv Nurs. 65 (2008) 149–160 https://doi.org/10.1111/j.1365-2648.2008.04852.x. J. Sadler, Effectiveness of student admission essays in identifying attrition. Nurse Educ Today. 23 (2003) 620–627 https://doi.org/10.1016/s0260-6917(03)00112-6. Y. Salamonson, S. Andrew, J. Clauson, M. Cleary, D. Jackson, S. Jacobs, Linguistic diversity as sociodemographic predictor of nursing program progression and completion. Contemp Nurse. 38 (2011) 84–93 https://doi.org/10.5172/conu.2011.38.1-2.84.
	Y. Salamonson, B. Everett, M. Cooper, L. Lombardo, R. Weaver, P.M. Davidson, Nursing as first choice predicts nursing program completion. Nurse Educ Today. 34 (2014) 127–131 https://doi.org/10.1016/j.nedt.2012.10.009. A. Wilson, A. Chur-Hansen, A. Marshall, T. Air, Should nursing-related work experience be a prerequisite for acceptance into a nursing programme? A study of students' reasons for withdrawing from undergraduate nursing at an Australian university. Nurse Educ Today. 31 (2011) 456–460 https://doi.org/10.1016/j.nedt.2010.09.005.
Data accessibility	With the article
Related research article	V. Caponnetto, A. Dante, V. Masotta, C. La Cerra, C. Petrucci, C.M. Alfes, L. Lancia, Examining nursing student academic outcomes: a forty-year systematic review and meta-analysis, Nurs Educ Today. https://doi.org/10.1016/j.nedt.2021.104823.

## Value of the Data

• These data contribute to understand nursing students’ academic outcomes occurrence and their associated factors.
• Researchers and educators of academic nursing students can benefit from these data to inform their future research and educational practice.
• These data might ease future literature summaries about the topic, they could be used to drive future research and compare their results with the literature available so far.

## 1 Data Description

**Fig. 1:**
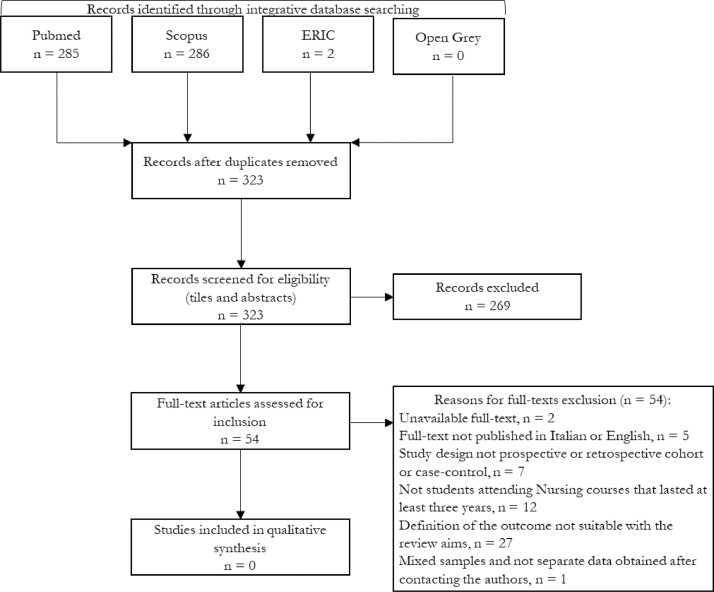
Integrative electronic search and selection strategy flow-chart.

In Table 1, all details about search strategy in electronic databases are reported, while Fig. 1 describes the output of the integrative electronic search conducted in 2020 and the selection strategy of retrieved references. The Supplementary file 1 lists all retrieved references through the three search strategies performed (i.e., initial search described in Table 1, scanning of reference lists and citations of the included studies, and additional search launched in January 2020). The output of initial search and references and citations scanning has been already published [1].

**Table 1:** Search strategy in electronic databases.

Database	Search strings
PubMed	("students, nursing"[MeSH Terms] OR ("students"[All Fields] AND "nursing"[All Fields]) OR "nursing students"[All Fields] OR ("nursing"[All Fields] AND "students"[All Fields])) AND ("tooth attrition"[MeSH Terms] OR ("tooth"[All Fields] AND "attrition"[All Fields]) OR "tooth attrition"[All Fields] OR "attrition"[All Fields])
	("students, nursing"[MeSH Terms] OR ("students"[All Fields] AND "nursing"[All Fields]) OR "nursing students"[All Fields] OR ("nursing"[All Fields] AND "students"[All Fields])) AND academic[All Fields] AND failure[All Fields]
	("students, nursing"[MeSH Terms] OR ("students"[All Fields] AND "nursing"[All Fields]) OR "nursing students"[All Fields] OR ("nursing"[All Fields] AND "students"[All Fields])) AND ("student dropouts"[MeSH Terms] OR ("student"[All Fields] AND "dropouts"[All Fields]) OR "student dropouts"[All Fields])
	("students, nursing"[MeSH Terms] OR ("students"[All Fields] AND "nursing"[All Fields]) OR "nursing students"[All Fields] OR ("nursing"[All Fields] AND "students"[All Fields])) AND wastage[All Fields]
	("students, nursing"[MeSH Terms] OR ("students"[All Fields] AND "nursing"[All Fields]) OR "nursing students"[All Fields] OR ("nursing"[All Fields] AND "students"[All Fields])) AND withdrawal[All Fields]
	("students, nursing"[MeSH Terms] OR ("students"[All Fields] AND "nursing"[All Fields]) OR "nursing students"[All Fields] OR ("nursing"[All Fields] AND "students"[All Fields])) AND academic[All Fields] AND success[All Fields]
	("students, nursing"[MeSH Terms] OR ("students"[All Fields] AND "nursing"[All Fields]) OR "nursing students"[All Fields] OR ("nursing"[All Fields] AND "students"[All Fields])) AND ("achievement"[MeSH Terms] OR "achievement"[All Fields])
	("students, nursing"[MeSH Terms] OR ("students"[All Fields] AND "nursing"[All Fields]) OR "nursing students"[All Fields] OR ("nursing"[All Fields] AND "students"[All Fields])) AND ("retention (psychology)"[MeSH Terms] OR ("retention"[All Fields] AND "(psychology)"[All Fields]) OR "retention (psychology)"[All Fields] OR "retention"[All Fields])

Scopus	nursing students AND attrition
	nursing students AND academic failure
	nursing students AND student dropouts
	nursing students AND wastage
	nursing students AND withdrawal
	nursing students AND academic success
	nursing students AND achievement
	nursing students AND retention

ERIC	nursing students AND attrition
	nursing students AND academic failure
	nursing students AND student dropouts
	nursing students AND wastage
	nursing students AND withdrawal
	nursing students AND academic success
	nursing students AND achievement
	nursing students AND retention

Open Grey	nursing students AND attrition
	nursing students AND academic failure
	nursing students AND student dropouts
	nursing students AND wastage
	nursing students AND withdrawal
	nursing students AND academic success
	nursing students AND achievement
	nursing students AND retention

Raw data extracted from each study are reported in the Supplementary file 2 (Excel for Windows spreadsheets). These data were utilized to report collected information in tables and figures.

In Table 2 (Supplementary file 3), all extended data extracted from the included studies are reported, especially data related to the research question of the review (i.e., study aim, outcome definition, independent variables, statistical analyses, and results and conclusions).

**Table 2:** Evaluation of risk of bias in the included studies.

	Reporting										External validity				Internal validity – bias				Internal validity - confounding					Total	
Study ID	#1	#2	#3	#4	#5	#6	#7*	#8	Total/Max score	Total std. (%)	#9	#10⁎⁎	Total/Max score	Total std. (%)	#11	#12	Total/Max score	Total std. (%)	#13	#14	#15*	Total/Max score	Total std. (%)	Total/Max score	Total std. (%)
1	1	0	1	0	1	1	na	1	6/8	75.0	1	0	1/2	50.0	1	1	2/2	100.0	1	1	na	2/2	100.0	11/14	78.6
2	1	1	1	2	1	1	na	0	7/8	87.5	1	0	1/2	50.0	1	1	2/2	100.0	1	1	na	2/2	100.0	12/14	85.7
3	1	1	1	2	1	1	na	1	8/8	100.0	1	1	2/2	100.0	1	1	2/2	100.0	1	1	na	2/2	100.0	14/14	100.0
4	1	1	1	2	1	1	0	1	8/9	88.9	1	1	2/2	100.0	1	1	2/2	100.0	1	1	1	3/3	100.0	15/16	93.8
5	1	1	1	2	1	1	1	1	9/9	100.0	1	1	2/2	100.0	1	1	2/2	100.0	1	1	1	3/3	100.0	16/16	100.0
6	1	1	1	1	1	1	1	1	8/9	88.9	1	0	2/2	100.0	0	1	1/2	50.0	1	1	1	3/3	100.0	14/16	87.5
7	1	1	0	0	1	1	na	0	4/8	50.0	1	0	1/2	50.0	0	1	1/2	50.0	1	1	na	2/2	100.0	8/14	57.1
8	1	1	1	2	1	1	na	1	8/8	100.0	1	0	1/2	50.0	1	1	2/2	100.0	1	1	na	2/2	100.0	13/14	92.9
9	1	1	1	0	1	1	na	1	6/8	75.0	0	na	0/1	0.0	1	1	2/2	100.0	1	1	na	2/2	100.0	10/13	76.9
10	1	1	1	1	1	1	na	1	7/8	87.5	1	1	2/2	100.0	1	1	2/2	100.0	1	1	na	2/2	100.0	13/14	92.9
11	1	1	1	1	1	1	na	1	7/8	87.5	1	1	2/2	100.0	1	1	2/2	100.0	1	1	na	2/2	100.0	13/14	92.9
12	1	1	1	1	1	1	1	1	8/9	88.9	1	0	1/2	50.0	0	1	1/2	50.0	1	1	1	3/3	100.0	13/16	81.3
13	1	1	1	2	1	1	1	1	9/9	100.0	1	1	2/2	100.0	0	1	1/2	50.0	1	1	1	3/3	100.0	15/16	93.8
14	1	1	1	2	1	1	na	1	8/8	100.0	1	1	2/2	100.0	1	1	2/2	100.0	1	1	na	2/2	100.0	14/14	100.0
15	1	1	0	0	1	1	na	0	4/8	50.0	1	1	2/2	100.0	1	1	2/2	100.0	1	1	na	2/2	100.0	10/14	71.4
16	1	1	1	2	1	1	1	1	9/9	100.0	1	1	2/2	100.0	1	1	2/2	100.0	1	1	1	3/3	100.0	16/16	100.0
17	1	1	1	2	1	1	1	1	9/9	100.0	1	1	2/2	100.0	1	1	2/2	100.0	1	1	1	3/3	100.0	16/16	100.0
18	1	1	1	1	1	1	na	0	6/8	75.0	1	0	1/2	50.0	0	1	1/2	50.0	1	1	na	2/2	100.0	10/14	71.4

#1: Is the hypothesis/aim/objective of the study clearly described? (0 = No; 1 = Yes) - #2: Are the main outcomes to be measured clearly described in the Introduction or Methods section? (0 = No; 1 = Yes) - #3: Are the characteristics of the patients included in the study clearly described? (0 = No; 1 = Yes) - #4: Are the distributions of principal confounders in each group of subjects to be compared clearly described? (0 = No; 1 = Partially; 2 = Yes) - #5: Are the main findings of the study clearly described? (0 = No; 1 = Yes) - #6: Does the study provide estimates of the random variability in the data for the main outcomes? (0 = No; 1 = Yes) - #7: Have the characteristics of patients lost to follow-up been described? (0 = No; 1 =Yes) - #8: Have actual probability values been reported (e.g. 0.035 rather than <0.05) for the main outcomes except where the probability value is less than 0.001? (0 = No; 1 = Yes) - #9: Were the subjects asked to participate in the study representative of the entire population from which they were recruited? (0 = No; 0 = Unable to determine; 1 = Yes) - #10: Were those subjects who were prepared to participate representative of the entire population from which they were recruited? (0 = No; 0 = Unable to determine; 1 = Yes) - #11: Were the statistical tests used to assess the main outcomes appropriate? (0 = No; 0 = Unable to determine; 1 = Yes) - #12: Were the main outcome measures used accurate (valid and reliable)? (0 = No; 0 = Unable to determine; 1 = Yes) - #13: Were the patients in different intervention groups (trials and cohort studies) or were the cases and controls (case-control studies) recruited from the same population? (0 = No; 0 = Unable to determine; 1 = Yes) - #14: Were study subjects in different intervention groups (trials and cohort studies) or were the cases and controls (case-control studies) recruited over the same period of time? (0 = No; 0 = Unable to determine; 1 = Yes) - #15: Were losses of patients to follow-up taken into account? (0 = No; 0 = Unable to determine; 1 = Yes).

⁎ Items not considered for retrospective cohort and case-control studies.

⁎⁎ Item not considered for case-control studies. na = not applicable

Table 3 reports the evaluation of risk of bias in the included studies according to the Downs’ & Black instrument [3], that was customized as needed.

**Table 3:** Frequency of academic success and lack of success according to the outcome definitions.

ACADEMIC SUCCESS	Graduation within the regular duration of the program				
	Study ID	First author, year	Sample	Success students (*N*)	% of success students

	1	Brimble, 2015	418	308	73.7%
	3	Dante et al., 2011	117	81	69.2%
	4	Dante et al., 2013	145	91	62.8%
	5	Dante et al., 2015	120	84	70.0%
	6	Deary et al., 2003	168	141	84.0%
	10	Lancia et al., 2018	2278	1402	61.6%
	11	Mulholland et al., 2008	1808	1431	79.2%
	12^°^	Pitt et al., 2014	138	46	33.3%
	13^°^	Pitt et al., 2015			
	14	Pryjmachuk et al., 2008	1173	727	62.0%
	16	Salamonson et al., 2011	352	106	30.1%
	18	Wilson et al., 2011	101	67	66.3%
		Total	7032	4484	63.8%

	Graduation not considering the time spent				
	2	Byrd et al., 1999	278	197	71.0%
	14	Pryjmachuk et al., 2008	1173	841	71.7%
15	Sadler, 2003	236	193	81.8%
	Total	1687	1231	73.0%

LACK OF ACADEMIC SUCCESS	Voluntarily drop out or withdrawal				
	7	Eherenfield et al., 1997	2,102	120	5.7%
	8	Kevern et al., 1999	354	89	25.1%
	9	Knopke, 1979	236	63	26.7%
	12^+^	Pitt et al., 2014	138	49	35.5%
	13^+^	Pitt et al., 2015			
	14	Pryjmachuck et al., 2008	1,173	86	7.3%
	16	Salamonson et al., 2011	352	113	32.1%
		Total	4355	520	12.0%

	Continuous enrolment in the program				
	12*	Pitt et al., 2014	138	43	31.2%
	13*	Pitt et al., 2015			
	14	Pryjmachuck et al., 2008	1173	246	21.0%
	16	Salamonson et al., 2011	352	133	37.8%
	Total	1663	422	25.4%

^°,+,*^Studies reporting data on the same sample, but different variables regarding the academic outcome.

Table 4 reports aggregated results about frequency of academic success and lack of success according to the outcome definitions reported in the studies.

**Table 4:** Summary of the results of meta-analysis, assessment of heterogeneity considering the study design for subgroup analysis, and sensitivity analysis as regards the outcome ‘graduation within the regular duration of the program’.

Variables	*k*	Meta-analysis results: association	Heterogeneity	Study design as possible cause of heterogeneity	Sensitivity analysis
Students were native speaker of the language of the Country where the study was conducted	4	No	Substantial	Yes. In prospective cohort studies the variable was significantly associated with the outcome, as opposed to retrospective cohort studies	Confirmed the meta-analysis results
Age	6	No	Substantial	No. In both the groups of prospective and retrospective cohort studies the variable was not associated with the outcome	Did not completely confirm the meta-analysis results
Female gender	8	Yes	Substantial	Yes. In both the groups of prospective and retrospective cohort studies the variable was significantly associated with the outcome	Confirmed the meta-analysis results
Students had attended Classical, Science, Academic, or Ordinary secondary schools	6	Yes	Absent	Subgroup analysis not needed	Confirmed the meta-analysis results
Higher secondary school grades	5	Yes	Substantial	No. In prospective cohort studies higher grades were significantly associated with the outcome, as opposed to retrospective cohort studies	Confirmed the meta-analysis results
Students have had work experience before attending the nursing program	2	No	Absent	Subgroup analysis not needed	Not feasible
Students have had work experience in the nursing field before attending the nursing program	3	No	Substantial	Not performed: it is overlapping with the sensitivity analysis	Confirmed the meta-analysis results
Students used to spend ≤ 30 min to reach the university with students	3	No	Absent	Subgroup analysis not needed	Confirmed the meta-analysis results
Students used to work while attending the nursing program	3	No	Absent	Subgroup analysis not needed	Confirmed the meta-analysis results
Weekly hours of work while attending the nursing program	2	No	Substantial	Subgroup analysis not feasible	Not feasible
Students used to volunteer while attending the nursing program	2	No	Absent	Subgroup analysis not needed	Not feasible
Students had experienced life events while attending the nursing program	2	No	Substantial	Subgroup analysis not feasible	Not feasible
Students had experienced economic hardship while attending the nursing program	2	No	Absent	Subgroup analysis not needed	Not feasible
Students had faced family commitments while attending the nursing program	2	No	Substantial	Subgroup analysis not feasible	Not feasible

**Fig. 2:**
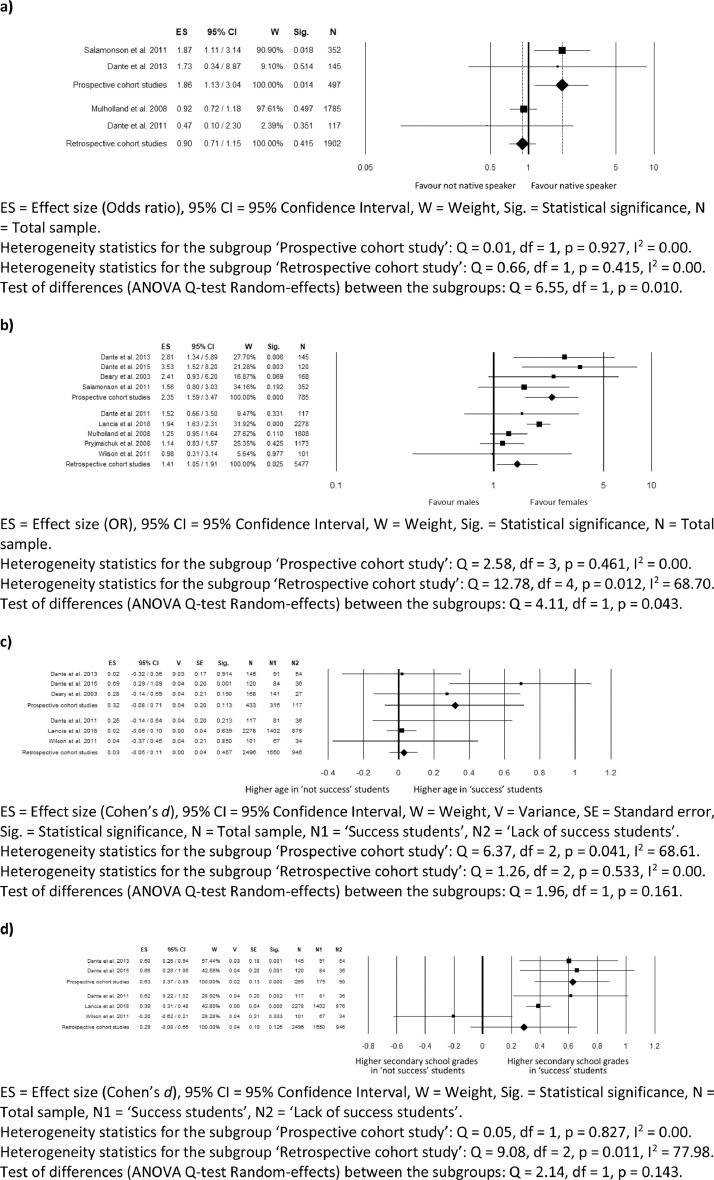
Subgroup analysis based on the study design for the meta-analysis comparing: (a) language (b) gender; (c) age; (d) secondary school grades.

**Fig. 3:**
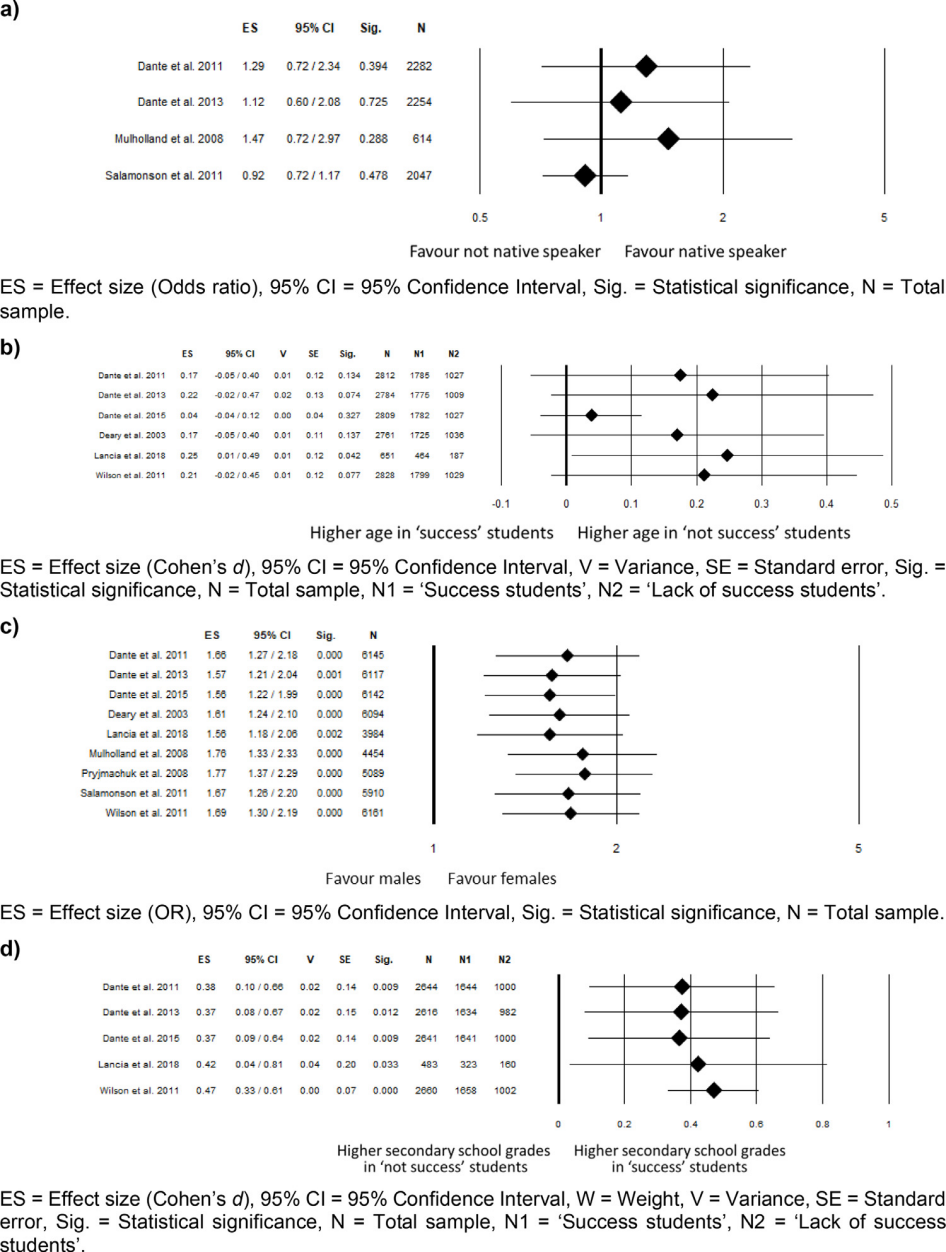
Sensitivity analysis for the meta-analysis comparing: (a) language; (b) age; (c) gender; (d) secondary school grades.

**Fig. 4:**
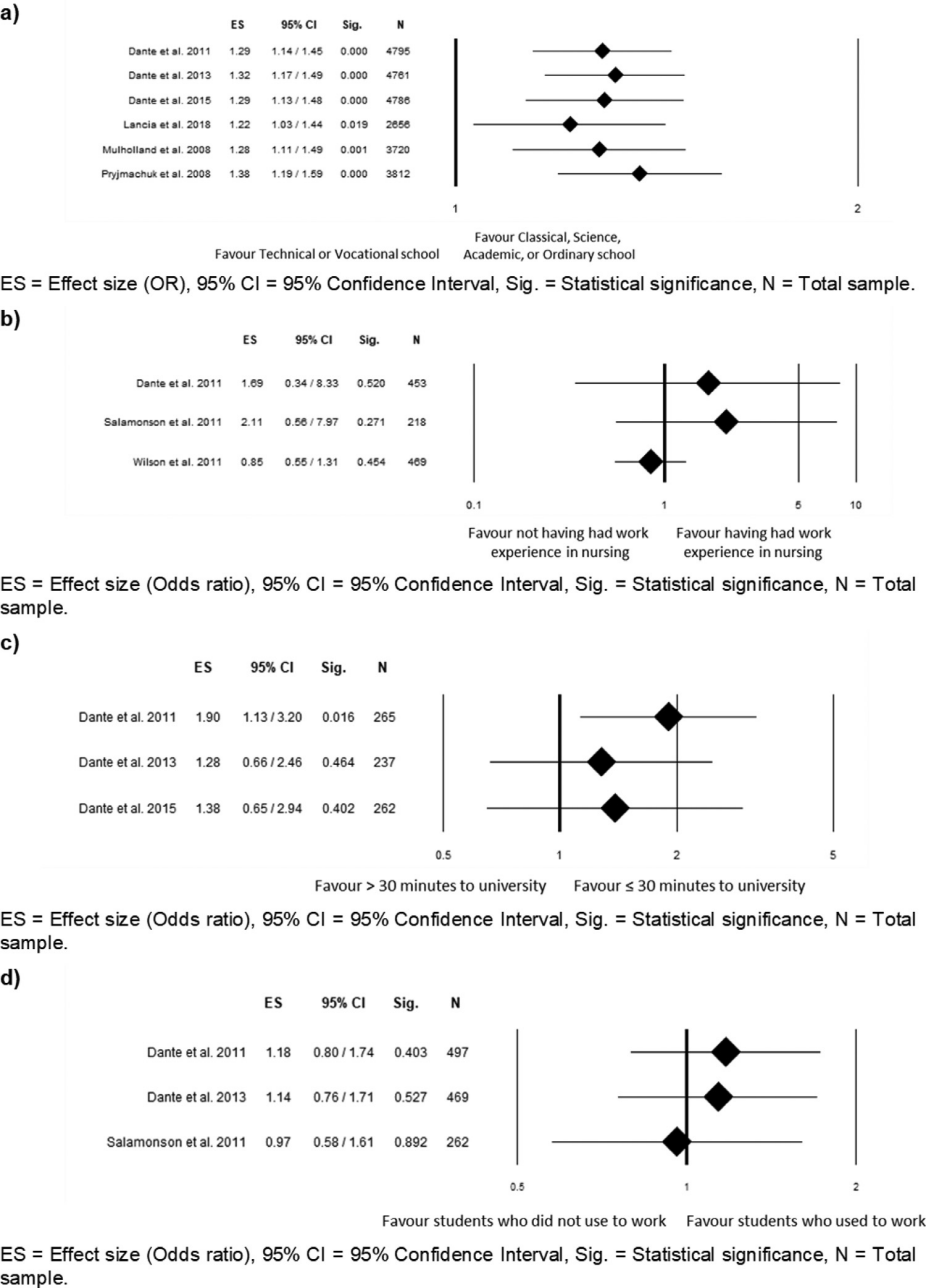
Sensitivity analysis for the meta-analysis comparing: (a) type of secondary school attended; (b) working experience in the nursing field before attending the nursing program; (c) time to reach the university; (d) working while attending the nursing program.

**Fig. 5:**
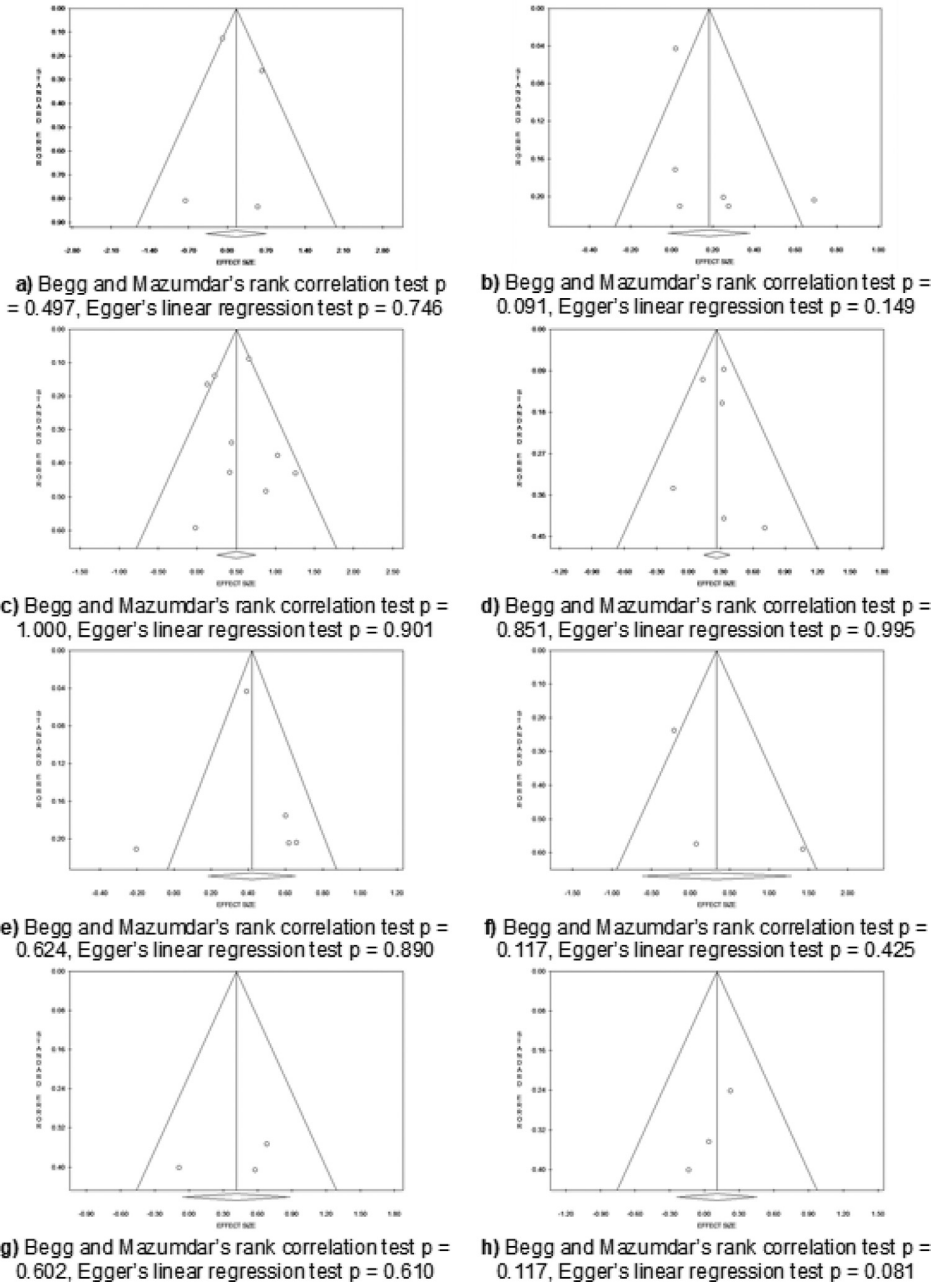
Funnel plots to assess the publication bias for the meta-analysis comparing: (a) language; (b) age; (c) gender; (d) type of secondary school; (e) secondary school grades;; (f) working experiences in the nursing field; (g) time spent to reach the university; (h) working while attending the nursing program.

Figures from 2 to 5 refer to the meta-analyses that considered as outcome nursing students' ‘graduation within the regular duration of the program’. In particular, Fig. 2 reports subgroup analyses based on the study design for the meta-analyses comparing language, gender, age, and secondary school grades; Fig. 3 reports sensitivity analysis for the meta-analyses comparing language, age, gender, and secondary school grades; Fig. 4 reports sensitivity analysis for the meta-analyses comparing type of secondary school attended, working experience in the nursing field before attending the nursing program, time to reach the university, and working while attending the nursing program; Fig. 5 reports funnel plots to assess the publication bias for the meta-analyses comparing language, age, gender, type of secondary school, secondary school grades, working experiences in the nursing field, time spent to reach the university, and working while attending the nursing program.

Finally, Table 4 reports a summary of the results of meta-analyses, assessment of heterogeneity considering the study design for subgroup analysis, and sensitivity analysis as regards the outcome ‘graduation within the regular duration of the program’.

## 2 Experimental Design, Materials, and Methods

An extensive systematic research and meta-analysis were performed in accordance with relevant criteria described in the ‘Cochrane Handbook for Systematic Reviews of Interventions’, Version 5.1.0 [2] and their reporting was checked against relevant items of the ‘Preferred Reporting Items for Systematic Reviews and Meta-Analyses’ (PRISMA) checklist [4].

To identify suitable keywords for the search strategy, a pilot search was performed in Scopus using the following search strings: a) nursing students and attrition; b) nursing students and retention; c) nursing students and dropout, along with the filter ‘Review’. Therefore, considering the keywords used in the retrieved reviews [5], [6], [7], [8], [9], [10], [11] and the aims of the present study, the following keywords were utilized in the search strategy: ‘students, nursing’, ‘achievement’, ‘academic success’, ‘retention’, ‘attrition’, ‘wastage’, ‘academic failure’, ‘student dropouts’, and ‘withdrawal’. The consulted electronic databases were PubMed, Scopus, Education Resources Information Center (ERIC), and Open Grey. The identified keywords were combined both for the research about academic success and failure. In regard to academic success, the following combinations were used: (a) students, nursing and achievement; (b) students, nursing and academic success; (c) students, nursing and retention. Instead, for the research about academic lack of success, the following strings were used: (a) students, nursing and attrition; b) students, nursing and wastage; (c) students, nursing and academic failure; (d) students, nursing and student dropouts; (e) students, nursing and withdrawal. The research was performed on January 21st, 2019 (limited to December 31st, 2018) and updated on January 24th, 2020 (limited from January 1st, 2019 to December 31st, 2019); no further limits or filters were utilized to ensure a high sensitivity of the search strategy and adopt a ‘broad approach’ [2]. All the retrieved references were collected and managed with EndNote X7.8 for Windows (Thomson Reuters, New York). Moreover, the reference lists of the included studies and references that had cited the included studies were retrieved through Scopus and assessed for eligibility.

To be included in the review, studies had to be observational in nature; undergraduate nursing students attending an academic program lasting at least three years were the considered population; all the measures of academic success and lack of success measured at least at the end of the regular duration of the nursing program were considered as outcomes.

Studies were screened for eligibility and inclusion analysing titles/abstracts and full-texts, respectively. Two raters independently screened titles and abstracts of the retrieved references. To avoid the exclusion of potentially relevant articles, in this phase, titles and abstracts had to fulfil the following broad criteria: (a) include any kind of nursing students or medical faculties; (b) describe the assessment of academic outcomes, even though generically defined as ‘achievement’.

Full-texts were analysed by two raters for their inclusion in the review considering the following as inclusion criteria: (a) the full-text was available through the library resources of the University of L'Aquila; (b) the full-text was published in Italian or English; (c) the full-text described quantitative non-randomized studies, i.e., the study design, assessed through the ‘List of study design’ [2], revealed to be prospective or retrospective cohort or case-control; (d) the sample of the study included academic nursing students that attended a program that lasted at least three years; (e) the authors had considered one of the definitions described in the literature regarding academic success or lack of success as outcomes and was measured at least at the end of the regular duration of the program; (f) the authors described the assessment of predictive or associated factors with the outcome. In this phase, the following exclusion criteria were considered: mixed samples (e.g., nursing and midwifery students) and not separate data available or obtained after contacting the authors. Finally, whether the same data were duplicated in different journals or included in larger and mixed samples, the paper presenting the highest methodological quality and most of data regarding nursing students was included. Studies that reported data on the same sample but about different variables regarding the academic outcome were included and treated as a unique study for descriptive statistics of the students and their characteristics, while they were considered as separate studies when assessing the results on associated or predictive factors. The whole selection and computing processes for the PRISMA flow-chart were performed using SPSS version 25.0 (IBM Corp., Armonk, NY, USA). Both in the eligibility and inclusion stages, the agreement among the judgements of the raters (inter-rater reliability) was estimated with the Krippendorff's alpha coefficient (α) ranging from 0 (totally disagree) to 1 (totally agree) [12]. Any disagreement between the raters was resolved by discussion with a third author until consensus was reached. The assessment of risk of bias was performed through the ‘Downs and Black instrument’ [3] after having modified it as needed. The following data were extracted: general information, study characteristics, and data related to the research question of the review. Authors were contacted if needed. Both risk of bias assessment and data extraction were performed by two raters independently and any disagreement was discussed with a third author. Descriptive data reported in the studies have been synthetized to provide an overview of the included studies and samples. Moreover, to detect the possible influence of the effects of micro-, meso-, and macro-level variables on the academic outcomes, data about each possible influencing variable were synthetized by pooling studies reporting the same definition of the outcome. When studies reported definitions of academic success and lack of success, investigating the associated/predictive variables of both definitions, results were first summarized referring to academic success as the outcome. Afterwards, when the definition of academic lack of success provided in these studies was not complementary to the definition of success (i.e., they referred to one aspect of lack of success such as failure), results were also summarized referring to academic lack of success as the outcome.

When more than two studies reporting the same definition of the outcome and influencing variable were retrieved, meta-analyses were performed utilizing the odds ratio (OR) or Cohen's *d* as effect sizes for categorical and continuous variables, respectively. The Random Effects Model (REM) was used to compute the meta-analyses through ProMeta free software. The Cochran's Q (χ^2^) and *I*^2^ were calculated for each meta-analysis for the assessment of heterogeneity. A subgroup analysis was conducted for the meta-analyses in which a ‘substantial’ or ‘considerable’ (i.e., *I^2^* ≥ 50%) heterogeneity was detected; a sensitivity analysis was performed for each meta-analysis that included three or more studies. For the meta-analyses that included three or more studies, the publication bias was assessed through funnel plots and tests for the asymmetry of the funnel plots (Begg and Mazumdar's rank correlation and Egger's linear regression method) [2]. Data that could not be included in the quantitative synthesis were narratively synthetized.

## Ethics statement

Not applicable.

## CRediT Author Statement

**Valeria Caponnetto** and **Angelo Dante:** Conceptualization, Methodology, Data analysis, Writing – original draft; **Vittorio Masotta** and **Carmen La Cerra:** Methodology, Data extraction, Software; **Loreto Lancia, Cristina Petrucci** and **Celeste Marie Alfes:** Conceptualization, Supervision, Writing – original draft.

## Declaration of Competing Interests

The authors declare that they have no known competing financial interests or personal relationships which have or could be perceived to have influenced the work reported in this article.

## Appendix Supplementary materials

Image, application 1

Image, application 2
