# Supplementary material for: Detailed data about a forty-year systematic review and meta-analysis on nursing student academic outcomes
Source: Data Brief. 2021 Aug 14;38:107298. doi: 10.1016/j.dib.2021.107298 (PMC8377560; doi:10.1016/j.dib.2021.107298)
Supplement: Supplementary file 2 [file mmc2.docx]

**Table 2. Data related to the research question of the review extracted from the included studies.**

| **Study ID: 1** | |
| --- | --- |
| **Aim** | To investigate the influence of the type of entry route on academic achievement |
| **Outcome definition** | Academic success: graduation within the regular duration of the program  *Lack of academic success: withdrawal* |
| **Independent variables** | Type of entry route, i.e. achieving a combination of A-levels that equate to 240 Universities and Colleges Admission Service (UCAS) points, an Access program pass, or a triple merit in a Business and Technology Education Council (BTEC) National Diploma |
| **Statistical analyses** | Not performed for the outcome of interest of the review |
| **Results and conclusions** | BTEC students appeared more likely to complete and Access entry route student least likely. Withdrawal from the program for personal reasons or discontinuation for academic failure is more likely in students who enter via the Access program route |
| **Study ID: 2** | |
| **Aim** | To determine the predictors of successful completion of the baccalaureate nursing program versus failure or dropout, as well as only versus failure |
| **Outcome definition** | Academic success: graduation during the period of the study  Lack of academic success: failure (second failure of a nursing course) and drop out |
| **Independent variables** | Model A - Success vs lack of success (failure and drop out): 1) age, ethnicity, previous baccalaureate degree, science GPA, social science GPA, and pre-nursing GPA (before enrollment) 2) age, ethnicity, previous baccalaureate degree, science GPA, social science GPA, pre-nursing GPA, and letter grades achieved in each nursing course (end of the first semester)  3) age, ethnicity, previous baccalaureate degree, science GPA, social science GPA, pre-nursing GPA, and letter grades achieved in each nursing course (end of the second semester) Model B - Success vs only failure:  1) age, ethnicity, previous baccalaureate degree, science GPA, social science GPA, and pre-nursing GPA (before enrollment)  2) age, ethnicity, previous baccalaureate degree, science GPA, social science GPA, pre-nursing GPA, and letter grades achieved in each nursing course (end of the first semester)  3) age, ethnicity, previous baccalaureate degree, science GPA, social science GPA, pre-nursing GPA, and letter grades achieved in each nursing course (end of the second semester) |
| **Statistical analyses** | Predictive model (logistic regression) |
| **Results and conclusions** | Model A - Success vs lack of success (failure and drop out): 1) age, ethnicity, science GPA, and pre-nursing GPA (before enrollment) showed a predictive power of 77.0% 2) age, pre-nursing GPA, and pharmacology grade (end of the first semester) showed a predictive power of 82.6% 3) age, ethnicity, social science GPA, and first medical-surgical course grade (end of the second semester) showed a predictive power of 90.9% Model B - Success vs only failure:  1) age, ethnicity, science GPA, and pre-nursing GPA (before enrolment) showed a predictive power of 87.8%  2) age, science GPA, pharmacology grade, and introduction to nursing grade (end of the first semester) showed a predictive power of 92.3% 3) ethnicity, pharmacology grade, introduction to nursing grade, first medical-surgical course grade, and fundamental of nursing grade (end of the second semester) showed a predictive power of 97.2% |
| **Study ID: 3** | |
| **Aim** | To document the factors associated with academic success or failure in an Italian cohort of nursing students |
| **Outcome definition** | Academic success: graduation within the regular duration of the program  *Lack of academic success: students who had not been awarded the required credits and continued to be enrolled and students who dropped out during the three years* |
| **Independent variables** | Age, gender, nationality, place of residence, distance of faculty from home, ranking in BNS entry exam, type of secondary school diploma, upper-secondary diploma grade, previous and concurrent work experience to the BNS, voluntary work while on BNS program, eventual number of weekly hours of work, eventual previous university experience in other fields, number of exams failed on theoretical courses, number of failure at the annual practical clinical assessment, family commitments during the BNS, life events experienced while studying for the BNS, economic difficulties perceived while studying for the BNS, intention to leave the BNS program |
| **Statistical analyses** | Association (t-test and Chi-square test) and predictive model (logistic multivariate regression) |
| **Results and conclusions** | Factors associated academic success were higher final grades awarded in secondary education and higher ranking awarded in BNS entry exam. Moreover, as regards the factors present during the BNS program, those associated with academic success were working less than 16 hours/week, not having had family commitments, not having had learning difficulties, having never failed the annual practical clinical assessment, not having had intention to leave the program.  Multivariate analysis identified two factors determining academic success/ lack of success: good results in the entry examination for the bachelor's degree in nursing sciences were associated with academic success, while family commitments were associated with lack of academic success |
| **Study ID: 4** | |
| **Aim** | To offer a dynamic analysis of the individual factors affecting student academic failure factors and investigating the predictive power of individual variables in the academic failure |
| **Outcome definition** | Academic success: graduation within the regular duration of the program  *Lack of academic success: students who had failed to complete the bachelor’s degree requirements within a period of three years and continued to be enrolled and students who dropped out* |
| **Independent variables** | Gender, age at enrolment, nationality, place of residence, upper-secondary diploma grade, eventual previous university experience in other fields, previous work or other experiences, changes in place of residence during the BNS, life events experienced while studying for the BNS, economic difficulties perceived while studying for the BNS, voluntary work while on BNS program, eventual work experience during the BSN and eventual number of weekly hours of work, other experiences and family commitments during the BNS |
| **Statistical analyses** | Association (Chi-square test, U-Mann-Whitney test) |
| **Results and conclusions** | Female gender and higher upper-secondary diploma grades were associated with academic success |
| **Study ID: 5** | |
| **Aim** | To examine the effect of selected individual student variables and clinical learning environments as perceived by students on academic success |
| **Outcome definition** | Academic success: graduation within the regular duration of the program |
| **Independent variables** | Gender, age at enrolment, upper-secondary school attended, nursing as first choice for career, upper-secondary diploma grade, time daily spent to reach university, Clinical Learning Environment Scale, Supervision and Nurse Teacher Scale (CLES+T) score for first and second academic year |
| **Statistical analyses** | Association (Chi-square test) and predictive model (logistic regression) |
| **Results and conclusions** | Factors associated academic success were female gender and upper-secondary diploma grades.  As regards the predictive power of the variables, in addition to some individual factors, i.e. female gender and upper-secondary diploma grade, three factors related to the clinical learning experience affected academic success: supervisory relationship, pedagogical atmosphere, and commitment of the ward |
| **Study ID: 6** | |
| **Aim** | To investigate the personal factors that lead to attrition in nursing students |
| **Outcome definition** | Academic success: graduation within the regular duration of the program  Lack of academic success: drop out |
| **Independent variables** | Gender, age at enrolment, cognitive ability, personality, coping strategies, and psychological distress |
| **Statistical analyses** | Association (t-test, Chi-square test) and predictive model (logistic regression) |
| **Results and conclusions** | Both the association and predictive analyses showed that less conscientious and ‘agreeable’ students were more likely to discontinue than other students |
| **Study ID: 7** | |
| **Aim** | To identify factors that lead to attrition in nursing programs and gain insight into possible measures that need to be considered to reduce the attrition rate |
| **Outcome definition** | Lack of academic success: drop out |
| **Independent variables** | Gender, immigrant status, completion of the army service, English language level, GPA |
| **Statistical analyses** | Association (test not declared) |
| **Results and conclusions** | Some variables were significant associated with attrition: male gender, no army service, low English pre-admission grade, low first-year GPA, and low second-year GPA |
| **Study ID: 8** | |
| **Aim** | To identify the relationship between student characteristics, retention and academic achievement, as well as the role of organizational factors in retention and achievement |
| **Outcome definition** | Lack of academic success: drop out |
| **Independent variables** | Gender, age at enrolment, mode of entry, entry qualifications and details of students' branch, cohort and education centre |
| **Statistical analyses** | Association (Chi-square test) |
| **Results and conclusions** | None of the investigated variables resulted to be associated with the outcome |
| **Study ID: 9** | |
| **Aim** | To investigate the factors associated to attrition in nursing students |
| **Outcome definition** | Lack of academic success: drop out during the period of the study |
| **Independent variables** | First semester GPA, secondary school percentile rank, science subpart of the College Qualification Test (CQT), student’s perception of the learning style (Nursing Student Self-Disclosure Inventory - NSSDI), personality needs (Personality Preference Schedule - EPPS) |
| **Statistical analyses** | Association (test not declared) |
| **Results and conclusions** | Variables significantly associated with attrition were first semester GPA, secondary school percentile rank, science subpart of the CQT, and three personality traits, i.e. order, dominance, and aggression |
| **Study ID: 10** | |
| **Aim** | To investigate any relation between nursing students' academic success and its potential predictors |
| **Outcome definition** | Academic success: graduation within the regular duration of the program |
| **Independent variables** | Gender, age at enrolment, upper-secondary school attended, upper-secondary diploma grade, admission test score, grade of intermediate exams |
| **Statistical analyses** | Predictive model (logistic regression) |
| **Results and conclusions** | Female gender, having attended a classical or scientific upper-secondary school, and having a higher upper-secondary diploma grade are predictive of nursing students' academic success |
| **Study ID: 11** | |
| **Aim** | To explore the relationship between selected diversity variables and nursing students’ progression and attrition |
| **Outcome definition** | Academic success: graduation within the regular duration of the program  *Lack of academic success: failure (due to not achieving the required standards) and students who voluntarily dropped out* |
| **Independent variables** | Gender, age at enrolment, Country of birth, ethnicity, educational qualifications, visa status, application route, absence rates |
| **Statistical analyses** | Association (Chi-square test) and predictive model (binary logistic regression) |
| **Results and conclusions** | The variables associated with academic success were higher age, Ireland, Zimbabwe or other English-speaking countries as Country of birth, Irish or African American as ethnic group, higher entry qualification, and whether the student required a visa.  The predictive variables of success were higher age, Ireland, Zimbabwe or other English-speaking Countries as Country of birth, and female gender. |
| **Study ID: 12** | |
| **Aim** | To report on the personal qualities, program performance, behaviors and progression of a sample of nursing students over three years |
| **Outcome definition** | Academic success: graduation within the regular duration of the program  Lack of academic success: drop out and students continuing their enrolment |
| **Independent variables** | Entry Personal Qualities Assessment (PQA) |
| **Statistical analyses** | Predictive model (logistic regression) |
| **Results and conclusions** | Resilience was the only significant predictor of students' likelihood of completing the program over 3 years, showing that as students' resilience score increased by 1, their chance of completing the program rather than continuing on after 3 years of study increased by 3%. Resilience had no significant impact of on withdrawal in relation to completion. |
| **Study ID: 13** | |
| **Aim** | To explore entry critical thinking scores relationship to students' demographic characteristics, academic and clinical performance  and progression |
| **Outcome definition** | Academic success: graduation within the regular duration of the program  Lack of academic success: drop out and students continuing their enrolment |
| **Independent variables** | Entry critical thinking skills |
| **Statistical analyses** | Predictive model (logistic regression) |
| **Results and conclusions** | This study found a significant relationship between student's entry critical thinking scores and their ability to complete the program in three years. No significant relationship was identified between critical thinking scores and drop out. |
| **Study ID: 14** | |
| **Aim** | To identify the factors having an impact on student completion rates in a preregistration nursing program |
| **Outcome definition** | Academic success: graduation regular duration of the program or later  Lack of academic success: continued in their study and voluntarily drop out |
| **Independent variables** | Gender, age at enrolment, cohort, pursued specialty (branch), trust of allocation, ethnicity, level and type of the highest qualification on entry, original domicile |
| **Statistical analyses** | Association (Chi-square test) and predictive model (logistic regression) |
| **Results and conclusions** | Age and educational qualifications on entry, being male or coming from an African American/minority ethnic group increased the risk of discontinuation from a nursing program, whereas attending the child specialty branch, being female or already having a university degree reduced the risk. Pursuing the child branch specialty or having the lowest level entry qualifications increased the risk of resignation from a nursing program, whereas having intermediate-level entry qualifications reduced the risk. |
| **Study ID: 15** | |
| **Aim** | To provide additional knowledge about the effectiveness of admission essays in identifying those students who drop out of a baccalaureate nursing program |
| **Outcome definition** | Academic success: graduation  Lack of academic success: not completion |
| **Independent variables** | Mean grade point average on admission test and scores on admission essays |
| **Statistical analyses** | Association (t-test) |
| **Results and conclusions** | In the group of non-completer students, the mean grade point average on admission was almost identical to the completers; mean scores on the admission essays were significantly higher in the completers |
| **Study ID: 16** | |
| **Aim** | To prospectively assess the entry characteristics of students, attrition, progression and completion in an undergraduate nursing program over a three-year period |
| **Outcome definition** | Academic success: graduation within the regular duration of the program  *Lack of academic success: students who were still enrolled or continuing to study in the program and students who were no longer enrolled and had discontinued (involuntary or voluntary) from the program prior to completing program requirements* |
| **Independent variables** | Gender, age at enrolment, marital status, enrolment status, Country of birth, spoken languages, nursing experience, working during the BN program, eventual weekly worked hours, GPA |
| **Statistical analyses** | Association (Kruskal-Wallis test, Chi-square test) and predictive model (multivariate logistic regression) |
| **Results and conclusions** | Native English speakers and students with a higher GPA score were most likely to complete the program at the end of the three years; program completers at the end of three years had also engaged in less hours of paid work during the semester period.  However, the regression analysis revealed that of the sociodemographic factors examined, being a native English speaker was the only significant predictor of program completion |
| **Study ID: 17** | |
| **Aim** | To examine the relationship between nursing as the program of first choice on entry into a Bachelor of Nursing (BN) program, and program completion |
| **Outcome definition** | Lack of academic success: attrition at the six-year follow-up |
| **Independent variables** | Gender, age at enrolment, cultural background, working during the BN program and eventual weekly worked hours, nursing as first choice, type of enrolment, enrolment category |
| **Statistical analyses** | Association (Chi-square test) and predictive model (multivariate logistic regression) |
| **Results and conclusions** | At the six-year follow-up, students who had selected nursing as their first choice were more likely to have completed the program than those students who had not selected nursing as their first choice; there were no significant differences in attrition based on type of enrolment (part-time versus full-time) or students' enrolment category (local students versus international students).  Moreover, logistic regression revealed that male students, students who worked more than 16 h per week during semester, and students who indicated nursing was not their first choice at entry into the program were significantly more likely to have dropped out of the program by the six-year follow-up |
| **Study ID: 18** | |
| **Aim** | To examine the reasons why students withdrew from a Bachelor of Nursing degree |
| **Outcome definition** | Academic success: graduation within the regular duration of the program |
| **Independent variables** | Gender, age at enrolment, subjects taken during final year of secondary school, prior nursing experience, knowing a nurse previously, matriculation score, whether participants were from a private or government school, and scores from the entrance interview |
| **Statistical analyses** | Association (t-test and Chi-square test) |
| **Results and conclusions** | Academic success was significantly associated to having had prior nursing experience and having known a nurse |

Italicized outcomes indicate definitions of lack of academic success which are complementary to the definitions of academic success provided in the studies. Such studies were included only in the summary of results about academic success.
